# IgG Galactosylation status combined with *MYOM2*-rs2294066 precisely predicts anti-TNF response in ankylosing spondylitis

**DOI:** 10.1186/s10020-019-0093-2

**Published:** 2019-06-13

**Authors:** Jing Liu, Qi Zhu, Jing Han, Hui Zhang, Yuan Li, Yanyun Ma, Dongyi He, Jianxin Gu, Xiaodong Zhou, John D. Reveille, Li Jin, Hejian Zou, Shifang Ren, Jiucun Wang

**Affiliations:** 1 State Key Laboratory of Genetic Engineering, Collaborative Innovation Center for Genetics and Development, School of Life Sciences, Fudan University, Shanghai, China; 2 Institute of Arthritis Research, Shanghai Academy of Chinese Medical Sciences, Guanghua Integrative Medicine Hospital, Shanghai, China; 3 Department of Biochemistry and Molecular Biology, Key Laboratory of Glycoconjugate Research Ministry of Public Health, School of Basic Medical Sciences, Fudan University, Shanghai, China; 4 Ministry of Education Key Laboratory of Contemporary Anthropology, Department of Anthropology and Human Genetics, School of Life Sciences, Fudan University, Shanghai, China; 5 Division of Rheumatology and Clinical Immunogenetics, the University of Texas-McGovern Medical School, Houston, TX USA; 6 Division of Rheumatology, Huashan Hospital, Fudan University, Shanghai, China; 7 Human Phenome Institute, Fudan University, Shanghai, China; 8 Institute of Rheumatology, Immunology and Allergy, Fudan University, Shanghai, China

**Keywords:** Ankylosing spondylitis, IgG-gal ratio, *MYOM2-*rs2294066, TNF blocker, Drug response prediction

## Abstract

**Background:**

Tumor necrosis factor (TNF) blockers have a high efficacy in treating Ankylosing Spondylitis (AS), yet up to 40% of AS patients show poor or even no response to this treatment. In this paper, we aim to build an approach to predict the response prior to clinical treatment.

**Methods:**

AS patients during the active progression were included and treated with TNF blocker for 3 months. Patients who do not fulfill ASASAS40 were considered as poor responders. The Immunoglobulin G galactosylation (IgG-Gal) ratio representing the quantity of IgG galactosylation was calculated and candidate single nucleotide polymorphisms (SNPs) in patients treated with etanercept was obtained. Machine-learning models and cross-validation were conducted to predict responsiveness.

**Results:**

Both IgG-Gal ratio at each time point and differential IgG-Gal ratios between week 0 and weeks 2, 4, 8, 12 showed significant difference between responders and poor-responders. Area under curve (AUC) of the IgG-Gal ratio prediction model was 0.8 after cross-validation, significantly higher than current clinical indexes (C-reactive protein (CRP) = 0.65, erythrocyte sedimentation rate (ESR) = 0.59). The SNP *MYOM2*-rs2294066 was found to be significantly associated with responsiveness of etanercept treatment. A three-stage approach consisting of baseline IgG-Gal ratio, differential IgG-Gal ratio in 2 weeks, and rs2294066 genotype demonstrated the ability to precisely predict the response of anti-TNF therapy (100% for poor-responders, 98% for responders).

**Conclusions:**

Combination of different omics can more precisely to predict the response of TNF blocker and it is potential to be applied clinically in the future.

**Electronic supplementary material:**

The online version of this article (10.1186/s10020-019-0093-2) contains supplementary material, which is available to authorized users.

## Introduction

Ankylosing spondylitis (AS) is an immune-mediated inflammatory disorder of the spine and sacroiliac joints, which could lead to vertebral fusion (Taurog et al., 2016). Although there is still no cure for AS, TNF-blockers are effective in alleviating inflammation and reducing pain (Sieper & Poddubnyy, 2016). However, while the annual cost of TNF-blockers is large, up to 40% of AS patients have no or poor response to this treatment (Schabert et al., 2013; Sieper & Poddubnyy, 2017). It is therefore essential to find biomarkers to predict the responsiveness of TNF blockers in clinical practice, preferably before or at the start of TNF blocker treatment.

Multiple studies have examined associations of genetic biomarkers, especially *TNFA* promoter polymorphisms, with responsiveness to TNF blocker treatment (Tong et al., 2012; Song et al., 2015; Tong et al., 2013; Liu et al., 2016). However, none have shown any model powerful enough to precisely distinguish good responders from poor ones.

Immunoglobulin G (IgG) plays an important role in humoral immune response by binding to antigen and Fcγ receptors (FcγR). Immune cells activity can be regulated by the glycan attached to asparagine-297 in the Fc part of IgG (Arnold et al., 2007). It was reported that the proportion of IgG lacking galactose is significantly increased in patients with autoimmune diseases (Vuckovic et al., 2015). IgG galactosylation has been reported as a biomarker for immune activation and cancer type screening (de Jong et al., 2016; Ren et al., 2016). Therefore, IgG galactosylation change in different time points during TNF blocker treatment is a plausible breakpoint in the development of prediction biomarkers.

In this study, we aimed to develop an approach to precisely predict the response to TNF blocker treatment among AS patients by both assessing the quantitative changes in IgG galactosylation alone and in combination with AS associated SNPs.

## Methods

### Subjects

**Fig. 1:**
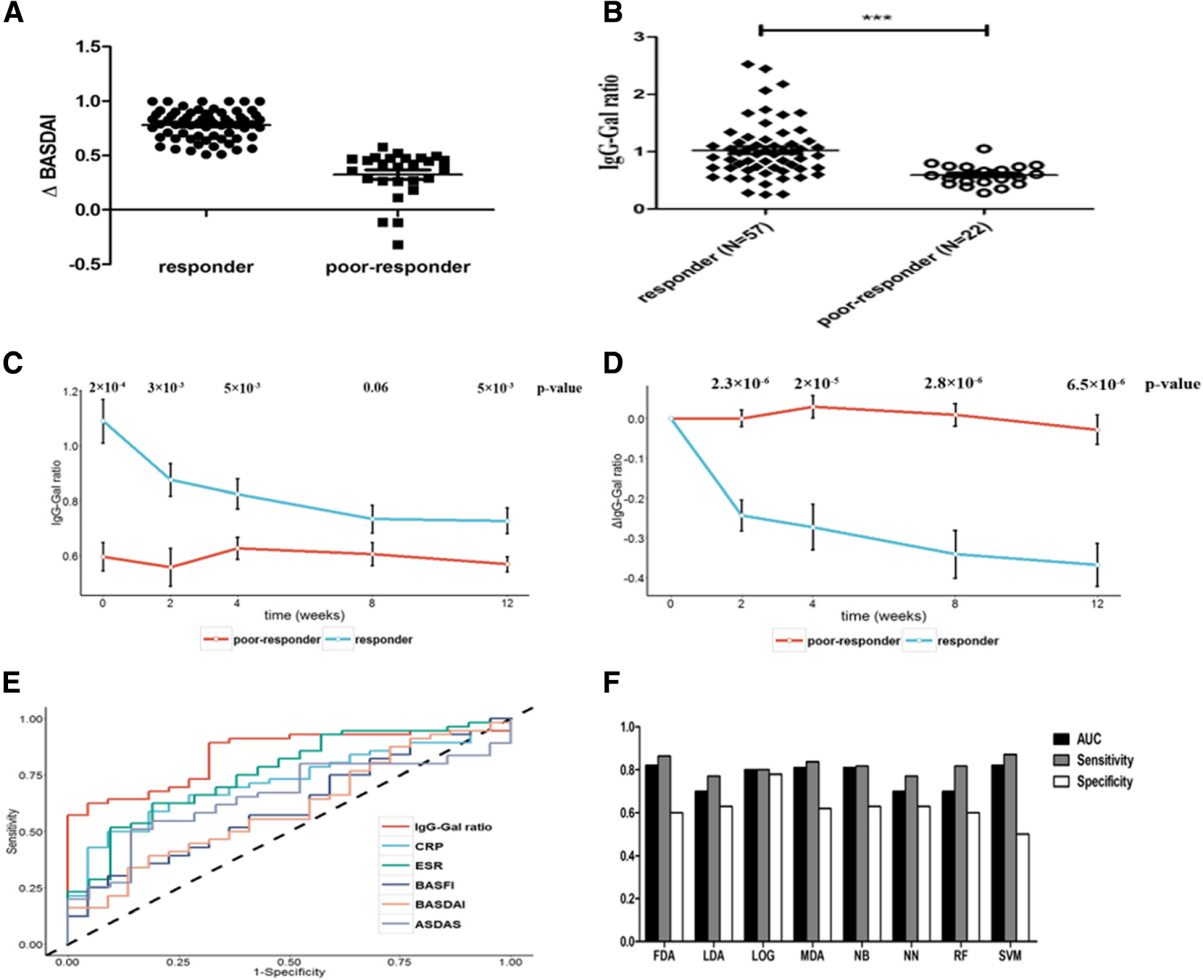
**a** Difference of ΔBASDAI between responders and poor-responders. ΔBASDAI = (BASDAI_week0_ – BASDAI_week12_)/BASDAI_week0._ **b** The difference of IgG-Gal ratio between responders and poor-responders before treatment. **c** IgG-Gal ratio variance of responders and poor-responders during treatment. **d** ΔIgG-Gal ratio variance of responders and poor-responders during treatment. The blank dots and black lines in C and D represent the mean value and standard error (se) interval respectively. **e** ROC curve of different indicators predicting patient response to etanercept. **f** Comparison between different models of patient response prediction. LG: logistic regression, RF: randomforest, SVM: support vector machine, NB: naviebayes, NN: neural network, LDA: linear discriminant analysis, MDA: mixture discriminant analysis, FDA: flexible discriminant analysis

Ninety-two AS patients expecting etanercept treatment were recruited from Guanghua hospital and Changhai hospital in Shanghai. Blood samples of the patients were collected at weeks 0, 2, 4, 8, and 12. Detailed information of the collected samples are described in Table 1. The inclusion criteria and exclusion criteria are summarized in Additional file 1: Table S1 and patients who did not fulfill ASAS40 were considered poor-responders. (Fig. 1a) (20, 21). The study was approved by the Ethical Committees of the School of Life Sciences of Fudan University and written and informed consent was obtained from each participant.

**Table 1:** The detailed information of AS patients

Parameter	Baseline (Mean ± SD, *N* = 79)	Week 2 (Mean ± SD, *N* = 79)	Week 4(Mean ± SD, *N* = 79)	Week 8 (Mean ± SD, *N* = 79)	Week 12 (Mean ± SD. *N* = 79)
Male n (%)	70 (89)				
age	36.0 ± 11.5				
HLA-B27 (%)	98.7				
disease duration (months)	7.3 ± 8.0				
NSAIDS (%)	82.3				
Psoriasis (%)	0				
Rheumatoid Arthritis (%)	0				
Inflammatory Bowel Disease	0				
BASDAI	5.4 ± 1.0	3.4 ± 1.5	2.2 ± 1.1	1.7 ± 1.0	1.4 ± 0.8
BASFI	3.2 ± 2.2	2.1 ± 1.8	1.4 ± 1.5	1.1 ± 1.2	0.9 ± 1.1
CRP	20.8 ± 32.2	3.8 ± 9.9	3.7 ± 10.0	3.1 ± 4.9	3.1 ± 4.2
ESR	28.3 ± 31.1	11.4 ± 19.7	8.4 ± 13.1	6.3 ± 6.8	6.5 ± 7.1
ASDAS	3.4 ± 0.8	1.8 ± 0.7	1.5 ± 0.7	1.2 ± 0.6	1.1 ± 0.7

*SD* Standard deviation, *BASDAI* Bath Ankylosing Spondylitis Di, *BASFI* Bath Ankylosing Spondylitis Fu, *ESR* Erythrocyte sedimentation rate, *CRP* C reactive protein, *ASDAS* Ankylosing Spondylitis Disease NSADIS Non-steroidal anti-inflammator

### Detection of IgG galactosylation ratio (IgG-gal ratio)

IgG-Gal ratio was detected by the method reported in our previous study. In brief, IgG N-glycans of purified IgG from blood plasma sample was released by mixing denatured IgG and PNGase F (New England Biolabs, Inc., USA) with incubation for 12 h at 37 °C. The released oligosaccharides were subsequently purified using PGC (porous graphitic carbon) and analyzed by AXIMA Resonance MALDI MS (Shimadzu Corp. JP) equipped with a 337 nm nitrogen laser in reflector positive ionization mode. Tandem mass spectrometry (MS/MS) was utilized to validate the component of the detected glycans. The GlycoWorkbench software was used for the annotation of MS spectra. Progenesis MALDI was used for MS data process. The biantennary, core-fucosylated structures carrying two, one, and no galactose residues are the three most significant glycans identified from IgG, named G2, G1, and G0. The distribution of IgG galactosylation (IgG-Gal ratio) was calculated using the relative intensities of G0, G1 and G2 according to the formula G0/ (G1 + G2 × 2).

### Whole-exome sequencing, data analysis, and genotyping

Whole-exome sequencing was performed on 33 (24 responders and 9 poor-responders) samples with an average coverage of 100× using Illumina HiSeq 2000. Burrows Wheeler Alignment V.0.7.10, and the genome analysis toolkit (GATK) V.1.1.28 were performed to map reads and call variants respectively against the reference sequences and Annovar was used for variant annotation.

Genotyping of all samples was performed by Sanger sequencing and candidate SNP was used to predict the response to the TNF blocker.

### Data analysis and cross-validation

The response to Etanercept of each patient was used as a dependent variable and several candidate indexes including Bath Ankylosing Spondylitis Di (BASDAI), Bath Ankylosing Spondylitis Fu (BASFI), Erythrocyte sedimentation rate (ESR), C reactive protein (CRP), Ankylosing Spondylitis Disease (ASDAS) and IgG-Gal ratio were used as independent variables to build the regression model. Cross-validation was applied as follows. The data of 78 patients were used to build the model and determine the best cutoff value; the value was used to predict the response of the 79th patient. The process was duplicated by 79 times.

As for the combination model of IgG-Gal ratio and candidate SNP, the methods logistic regression (LG), randomforest (RF), support vector machine (SVM), naviebayes (NB), neural network (NN), linear discriminant analysis (LDA), mixture discriminant analysis (MDA) and flexible discriminant analysis (FDA) were performed. Cross-validation was applied as follows: we randomly divided the responders and poor-responders into 10 groups; groups 1 to 8 were used to build model (training), and groups 9 and 10 were used to test the model (validation) and then rotate 10 times. All models were performed by R software with “randomForest”, “e1071”, “nnet”, “MASS” and “mda” packages.

ROC (receiver operating characteristic) curve was plotted and sensitivity as well as specificity were calculated to test the power of the index to predict patient response to Etanercept.

ROC curve was performed by “ggplot2” package in R.

## Results

### IgG-gal ratio is significantly different between responders and poor-responders

Etanercept is the most widely used type of TNF blocker for AS patients in China.. Plasma from 79 patients were collected at weeks 0, 2, 4, 8, and 12 during treatment for measuring IgG galactosylation level (IgG-Gal) (Table 1).

IgG-Galactosylation ratio (IgG-Gal ratio) was measured by the method reported in our previous study (Ren et al., 2016). In particular, IgG N-glycans of purified IgG was released by mixing denatured IgG and PNGase F. The released oligosaccharides were detected by tandem mass spectrometry. The biantennary, core-fucosylated structures carrying two, one, and none galactose residues are the three most common glycans identified from IgG, named G2, G1, and G0. The distribution of IgG galactosylation (IgG-Gal ratio) was calculating using relative intensities of G0, G1 and G2 according to the formula of G0/ (G1 + G2 × 2). (See details in the Additional file 1).

As depicted in Fig. 1b, at the beginning (week 0), the average IgG-Gal ratio of responders was almost twice as high as that of poor-responders (1.02 ± 0.49 vs 0.59 ± 0.17, *p*-value = 2 × 10^− 4^). After treatment, the average IgG–Gal ratio of responders decreased slowly from 1.1 to 0.8 (P_trend_ = 2.9 × 10^− 5^), while that of poor-responders persisted (P_trend_ = 0.82) (Fig. 1c). Furthermore, difference of IgG-Gal ratio between responders and poor-responders at each time-point showed at least marginal significance. We further calculated the difference of IgG-Gal ratios between week 0 and weeks 2, 4, 8, and 12 (ΔIgG-Gal ratio). The ΔIgG-Gal ratios displayed significant difference at each time point between responders (*n* = 42) and poor-responders (*n* = 10) (Fig. 1d). On the contrary, the indices such as BASDAI, BASFI, ESR, CRP and ASDAS had little or no significant difference between responders and poor-responders before treatment (Additional file 1: Figure S1). The BASDAI and BASFI (as well as the ASDAS in part) are considered subjective indexes largely influenced by human factors. The ESR and CRP were observed to decline sharply to a certain low level after two weeks of treatment, indicating sensitivity towards anti-inflammatory drugs. The five indexes mentioned above are therefore considered less reliable for the prediction of responsiveness towards etanercept treatment in AS patients.

### IgG-gal ratio predicts response to TNF blocker treatment

The IgG-Gal ratio was compared to current indexes in prediction of patient response to TNF blocker treatment using ROC curves, sensitivity and specificity. Results revealed that BASDAI and BASFI before treatment had little power to predict patient response. ROC curves showed that IgG-Gal ratio and ΔIgG-Gal ratio (Additional file 1: Figure S2) were best in prediction of patient response, compared to BASDAI, BASFI, ESR, CRP and ASDAS (Fig. 1e). The AUCs (area under curve) of IgG-Gal ratio and ΔIgG-Gal ratio were 0.83 and 0.85 respectively, higher than the others. The specificity of IgG-Gal ratio reached 0.95, significantly higher than those of BASDAI, BASFI, CRP, ESR and ASDAS (Additional file 1: Figure S1F). After cross-validation, results showed that the AUC value of IgG-Gal ratio was 0.80, also significantly higher than those of BASDAI, BASFI, ESR, CRP and ASDAS. The specificity of IgG-Gal ratio was also highest among all 6 indexes (Additional file 1: Table S2).

### *MOYM2* rs2294066 is associated with patient response to etanercept

Whole-exome sequencing of 24 responders and 9 poor-responders was performed. Six SNPs of Myomesin 2 (*MYOM2), Vacuolar Protein Sorting 13 Homolog B (VPS13B), Dispatched RND Transporter Family Member 1 (DISP1)* and *Interleukin (IL27)* genes were most significantly different between responders and poor-responders*.* After the validation stage (responder = 40, poor-responder = 19), only *MYOM2* SNP rs2294066 remained significant (*p*-value = 5.76E-04) (Table 2). The information of other five SNPs is presented in Additional file 1: Table S3. Patients carrying CC genotype were mainly responders, and about half patients with CT or CC genotype were responders (Table 2). The sensitivity, specificity and AUC value of *MYOM2* rs2294066 were 0.81, 0.58 and 0.70 respectively.

**Table 2:** Distribution of SNP (rs2294066) in *MYOM2* between responders and poor-responders

Genotype	CC (%)	CT (%)	TT (%)	*P* value
Responder	52 (81.3)	11 (17.2)	1 (1.5)	0.000576
Poor-responder	12 (42.9)	15 (53.6)	1 (3.5)	
Allele	C	T	P value	OR (95% CI)
Responder	115	13	0.0011	3.82 (1.59–9.42)
Poor-responder	39	17		

### Combination of IgG-gal ratio and *MYOM2* genotype precisely predicts patient response to etanercept

We combined IgG-Gal ratio and *MYOM2* (rs2294066) with 8 machine-learning models to achieve a more precise prediction of AS patient response to etanercept treatment.

After 10-times cross-validation, as depicted in Fig. 1f (for details please refer to the legend of Fig. 1f), the AUC values and the sensitivity of the FDA and SVM models were 0.82 and 0.87 respectively, higher than others were. The specificity of the LG model was highest (0.78). The plot revealed that the AUCs of all models in predicting patient response to etanercept were approximately 0.8, suggesting that combination of IgG-Gal ratio and *MYOM2* rs2294066 to be a reliable predictor.

### The three-stage approach is a potentially applicable method in prediction of etanercept response in clinical practice

It is of great importance to screen patients and separate responders from non-responders. IgG-Gal ratio prior to treatment, ΔIgG-Gal ratio (IgG-Gal ratio_week2_ – IgG-Gal ratio_week0_) and *MYOM2* rs2294066 were used to differentiate responders from non-responders in each stage respectively in clinical practice.

**Fig. 2:**
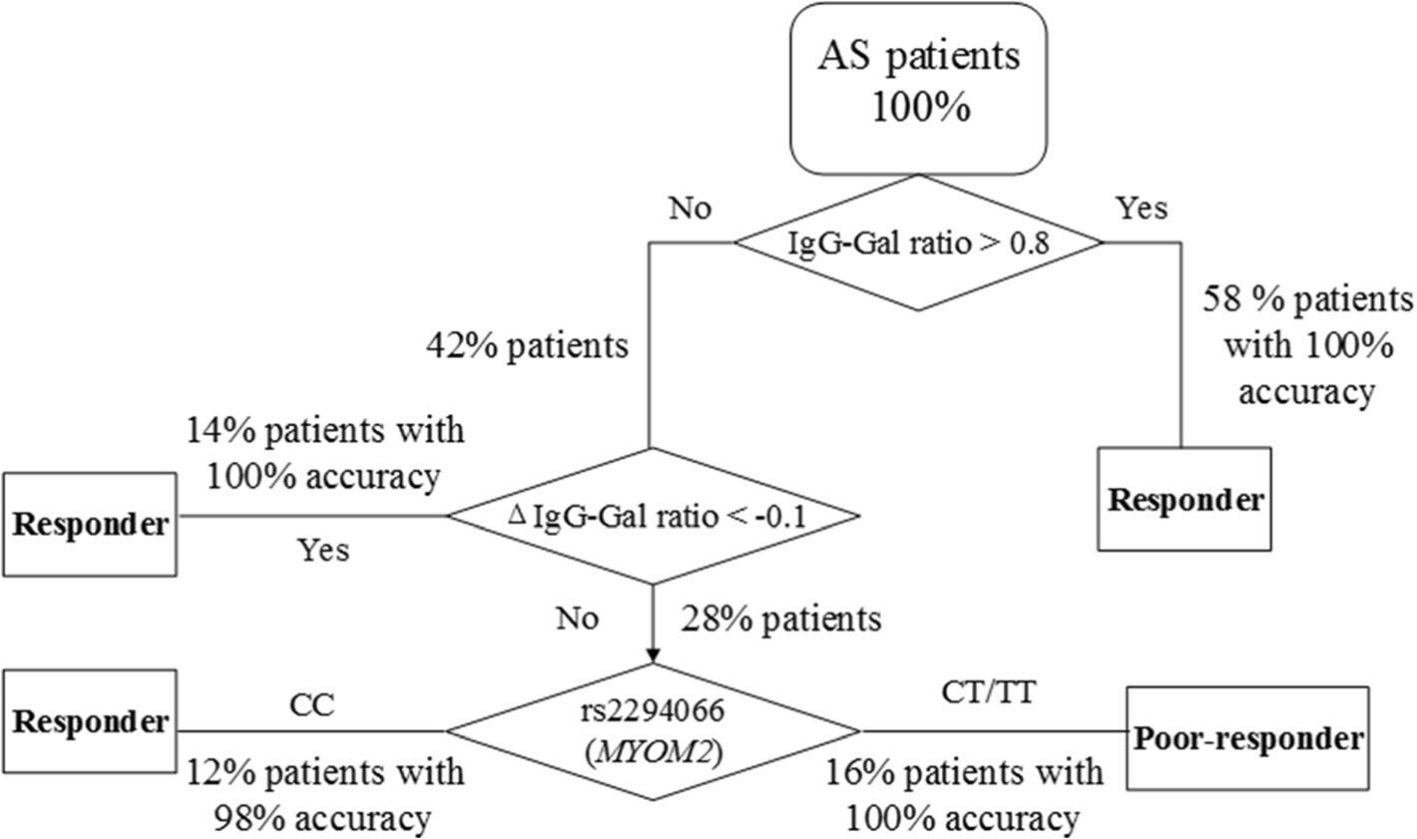
Flowchart of the novel three-stage method in prediction of etanercept patient response. IgG-Gal ratio: IgG galactosylation ratio, ΔIgG-Gal ratio: The difference of IgG-Gal ratio between weeks 0 and 2

Flowchart of the three-stage method in prediction of etanercept patient response is depicted in Fig. 2.

#### Stage I

The IgG-Gal ratio of all AS patients was evaluated prior to TNF-blocker treatment. 58% of all patients whose IgG-Gal ratio value was over 0.8 were predicted to be responders with 100% accuracy.

#### Stage II

42% of all patients with IgG-Gal ratio values under 0.8 were treated with one dose of etanercept and repetitive IgG-Gal ratio evaluation was performed at week 2, among which 14% of the total patients whose ΔIgG-Gal ratio value were below − 0.1 were predicted to be responders with 100% accuracy.

#### Stage III

Patients with IgG-Gal ratio values under 0.8 and ΔIgG-Gal ratio values above − 0.1 accounted for 28% of all patients. They were subjected to *MYOM2* rs2294066 genotyping, revealing 16% of all patients with CT/TT genotype to be poor-responders with 100% accuracy and 12% of all patients with CC genotype to be responders with 98% accuracy.

## Discussion

Genetics and environment are two important factors to the pathogenesis of AS (Sieper & Poddubnyy, 2017). TNF blockers target to TNFα, which participates in complex inflammatory pathways. Therefore, in order to predict the response of TNF blocker precisely, we should take both genetic and environmental factors into consideration. In this article, we combined the data of IgG-Gal ratio, a typical intracellular environmental factor, and *MYOM2* (rs2294066) to predict the response of TNF blocker.

Glycan modification plays an important role in autoimmune diseases (Mesko et al., 2012; Theodoratou et al., 2014). In our previous studies, we have exploited a method to assess the alteration of serum IgG galactosylation, which has great stability and can detect the galactosylation level of each individual quantitatively (Ren et al., 2016), and thus we applied it here to explore the potential IgG glycan predictors for responsiveness to the TNF blocker in AS patients for the first time. In addition, ESR and CRP declined sharply to a certain low level after two-week treatment indicating that these two clinical indexes are largely influenced by anti-inflammatory drug, and thus they are not reliable biomarkers. However, The IgG-Gal ratio showed significant difference between responders and non-responders at any time point during the treatment which indicated that it was a stable biomarker.

Because patients with different genetic background should have different response to drugs and previous researches found pharmacogenomics was a good approach in predicting the response to the TNF blocker in immune rheumatism (Liu et al., 2016; Salgado et al., 2014; Billiet et al., 2016; Cuchacovich et al., 2014). Therefore, we performed whole-exome sequencing to find associated SNPs. We found that SNP in MYOM2 was associated with response of TNF blocker and patients with T allele had higher *MYOM2* gene expression (Additional file 1: Figure S3). It is a missense variant in coding region (Thr776Met) and had relatively high sensitivity in screening responders from AS patients. Previous studies have reported that over expression of EH-myomesin (*MYOM2* isoform) served as a biomarker for dilated cardiomyopathy and TNF-α was up-regulated during brief myocardial ischemia, or coronary occlusion (Zingarelli et al., 2002; Kimura et al., 2006). It indicated that MYOM2 might associated with the pharmacology of TNF blockers.

Therefore, we proposed a novel three-stage approach to combine genetic markers and post-translational modifications to predict precisely the response of the TNF blocker etanercept in AS patients. Briefly, when an AS patient is considered to be treated with TNF blocker, first we detect IgG-Gal ratio and if it is over 0.8 we advise him to be treated with TNF blocker for long-term. If it is below 0.8, we suggest him to be treated with TNF blocker for one time and IgG-Gal ratio will be detected again after two weeks. If ΔIgG-Gal ratio is below − 0.1, he can be treated with TNF blocker for long-term, or he should further be detected the genotype of *MYOM2* (rs2294066). If he carries CT/TT genotype he will be advised to change to another treatment. If he carries CC genotype, we suggest him to continue the treatment with TNF blocker.

The limitation of this study was that it only included the result from single medical center and the sample number is not very large. Therefore, in the future the result will be further validated in another medical center.

## Conclusions

In this study, in order to predict the response of TNF blocker more precisely, we combined genetic data with protein modification data. This was a pilot study, but it largely increased the power of prediction. We propose that it is a potential biomarker to combine different omics data in the future.

## Additional file

Additional file 1: **Figure S1.** A: The variance of BASFI with time course. **Figure S2.** A: ΔIgG-Gal ratio of responders and poor-responders after IgG-Gal ratio screening. **Figure S3.** *MYOM2* gene expression of different alleles (Wilcoxon test). **Table S1.** Inclusion criteria. **Table S2.** Mean value of sensitivity, specificity and AUC of different indexes after cross-validation. **Table S3.** Validation results of the other 5 SNPs that were associated with the response to TNF blocker in the first stage. (DOCX 886 kb)

## Data Availability

The datasets used and/or analysed during the current study are available from the corresponding author on reasonable request.

## Abbreviations

AS: Ankylosing spondylitis
AUC: Area under curve
FcγR: Fcγ receptors
FDA: Flexible discriminant analysis
IgG: Immunoglobulin G
IgG-Gal ratio: IgG-Galactosylation ratio
LDA: Linear discriminant analysis
LG: Logistic regression
MDA: Mixture discriminant analysis
NB: Naviebayes
NN: Neural network
RF: Randomforest
SVM: Support vector machine
