# Supplementary material for: IgG Galactosylation status combined with MYOM2-rs2294066 precisely predicts anti-TNF response in ankylosing spondylitis
Source: Mol Med. 2019 Jun 13;25:25. doi: 10.1186/s10020-019-0093-2 (PMC6567531; doi:10.1186/s10020-019-0093-2)
Supplement: Supplementary file 1 — Figure S1. A: The variance of BASFI with time course. Figure S2. A: ΔIgG-Gal ratio of responders and poor-responders after IgG-Gal ratio screening. Figure S3. MYOM2 gene expression of different alleles (Wilcoxon test). Table S1. Inclusion criteria. Table S2. Mean value of sensitivity, specificity and AUC of different indexes after cross-validation. Table S3. Validation results of the other 5 SNPs that were associated with the response to TNF blocker in the first stage. (DOCX 886 kb) [file 10020_2019_93_MOESM1_ESM.docx]

**IgG Galactosylation Status and MYOM2-rs2294066 can Precisely Predict anti-TNF Response**

A B


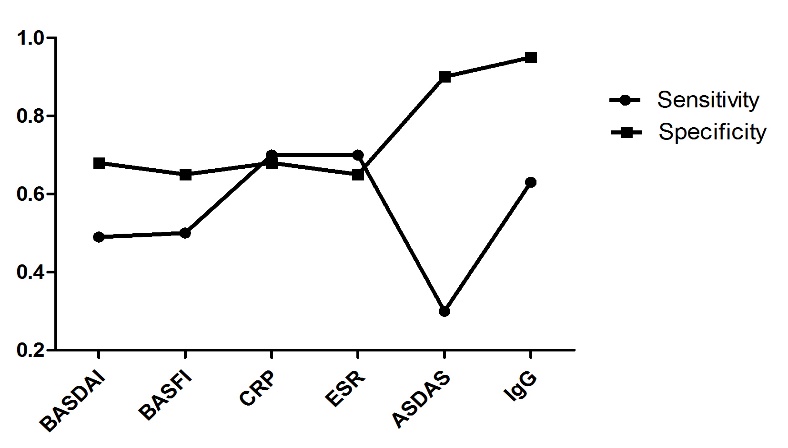

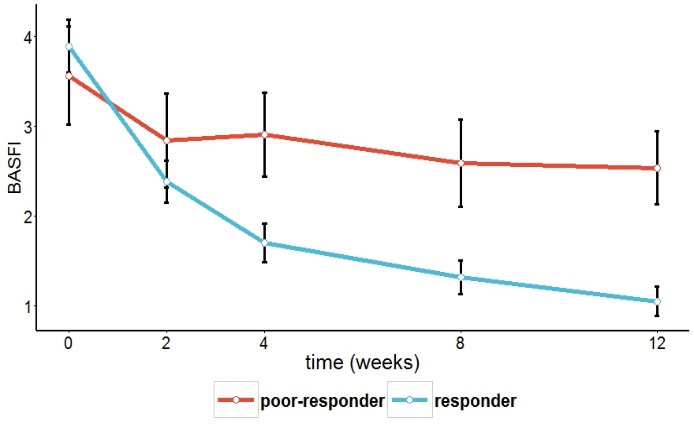

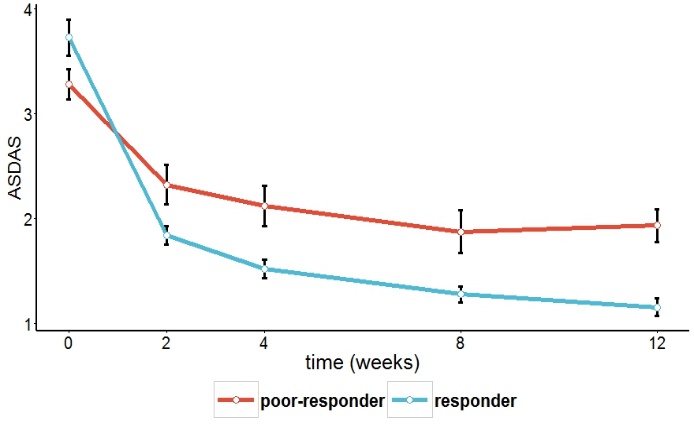

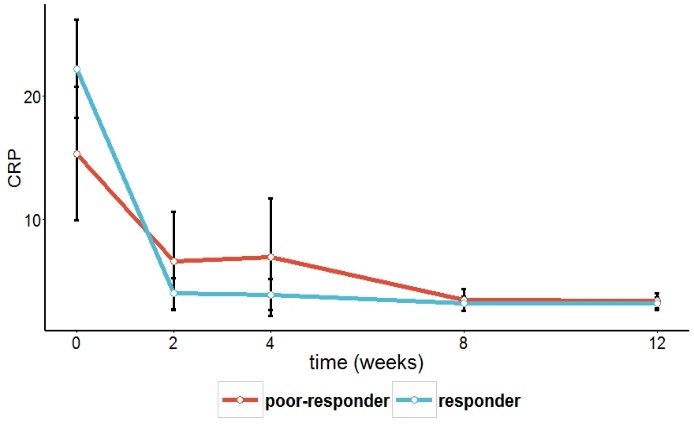

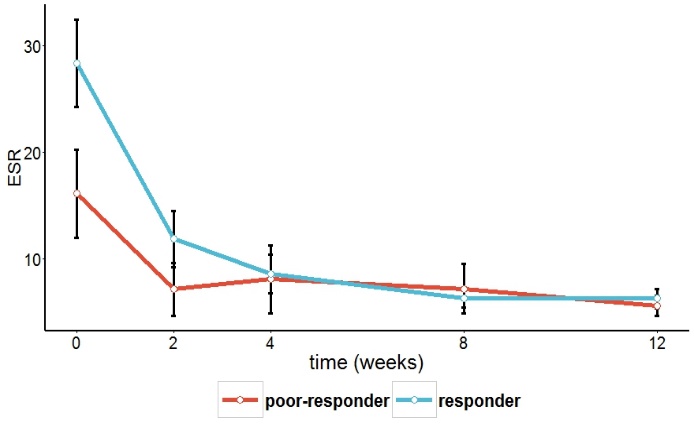

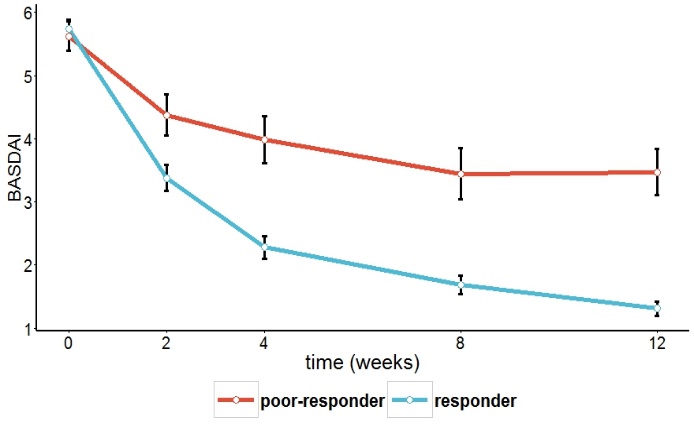


C D

E F

Figure S1. A: The variance of BASFI with time course

B: The variance of ASDAS with time course

C: The variance of CRP with time course

D: The variance of ESR with time course

E: The variance of BASDAI with time course

F: Sensitivity and specificity values of different indexes

A

B


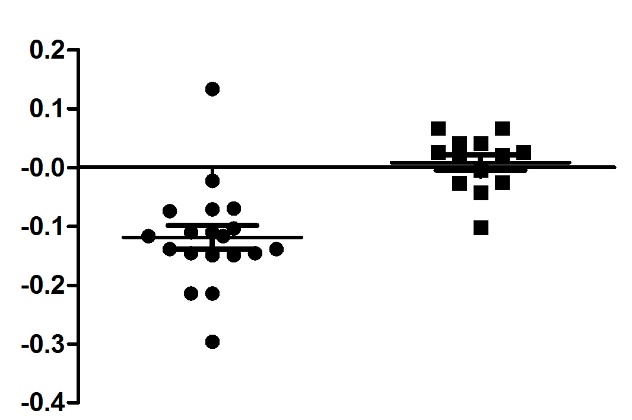

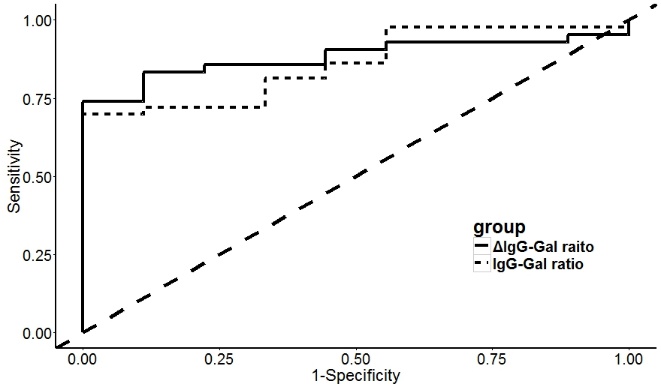


Figure S2. A: ΔIgG-Gal ratio of responders and poor-responders after IgG-Gal ratio screening.

B: ROC curve of ΔIgG-Gal ratio and IgG-Gal ratio in predicting patient response to Etanercept..

Patients in the circle (58% AS patients) were predicted to be responders with 100% accuracy.

ΔIgG-Gal ratio: the difference of IgG-Gal ratio between weeks 0 and 2.


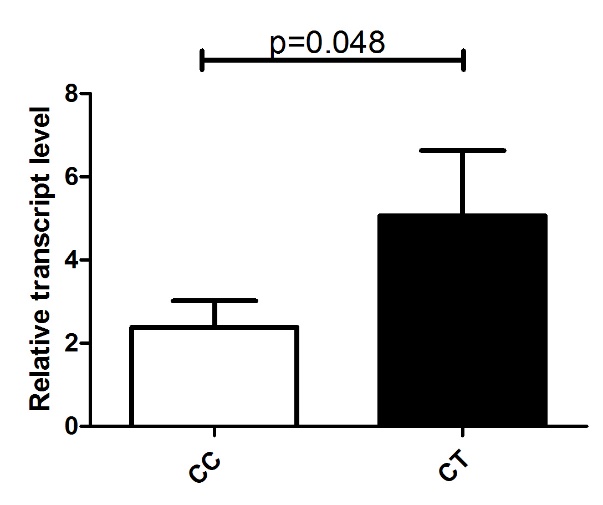


Figure S3. *MYOM2* gene expression of different alleles (Wilcoxon test)

Number of CC allele: 35, number of CT allele: 15

Table S1. Inclusion criteria

| **Inclusion criteria** |
| --- |
| 1. age between 18-65yrs |
| 2. capable of understanding the content and procedure of the experiment; signed informed consent |
| 3. fulfilles the 1984 modified New York criteria and new ASAS classification criteria for peripheral SpA |
| 4. BASDAI ≥4 and back pain ≥4 |
| 5. history of NSADIs treatment for ≥4 weeks, with poor response |
| 6. has undergone stable NSADIS treatment for ≥2 weeks |
| 7. glucocorticoid dosage stably ≤10 mg/day or termination of the use of glucocorticoids for 12 weeks |
| 8. stable dosage of DMARDs for 4 weeks or termination of the use of DMARDs for 4 weeks |
| 9. lab examinations fulfill the following |
| hemoglobin ≥85 g/L |
| 3.5×10^9^/L ≤ White blood cell count ≤ 10×10^9^/L |
| platelet ≥ lower limits of normal |
| Liver function (ALT, Tbil) ≤ twice of upper limits of normal |
| kidney function ≤ upper limits of normal |
| 10. participates can be followed-up |
| **Exclusion criteria** |
| 1. Allergic to TNF or other medications. 2. Once used TNF blocker for more than 3 months, but it has no response 3. Once was infected with tuberculosis 4. Patients were in infection period 5. HBs-Ag or HCV-Ab positive |
| 1. Once was infected with other autoimmune diseases 2. Once was infected with cancer |
|  |
|  |
|  |
|  |
|  |
|  |

Table S2. Mean value of sensitivity, specificity and AUC of different indexes after cross-validation

| clinical index | sensitivity | specificity | AUC^a^ |
| --- | --- | --- | --- |
| BASDAI | 0.54 | 0.45 | 0.50^***^ |
| BASFI | 0.79 | 0.23 | 0.51^***^ |
| CRP | 0.75 | 0.54 | 0.65^*^ |
| ESR | 0.60 | 0.60 | 0.59^**^ |
| ASDAS | 0.30 | 0.67 | 0.52^***^ |
| **IgG** | **0.68** | **0.86** | **0.80** |

^a^ p-value was calculated between IgG-Gal ratio and other indexes (*: p-value < 0.05, **: p-value < 0.01, ***: p-value < 0.001)

Table S3. Validation results of the other 5 SNPs that were associated with the response to TNF blocker in the first stage.

| allele | responder | poor-responder | Odds ratio | p-value |
| --- | --- | --- | --- | --- |
| *MYOM2* (rs968381) | | |  |  |
| A | 87 | 28 | 1.95 | 0.07 |
| G | 35 | 22 |  |  |
| *VPS13B* (rs7460625) | | |  |  |
| G | 67 | 29 | 0.89 | 0.69 |
| T | 57 | 27 |  |  |
| *DISP1* (rs2609383)* | | |  |  |
| G | 91 | 34 | 1.2 | 0.87 |
| A | 31 | 14 |  |  |
| *DISP1* (rs2789975)* | | |  |  |
| G | 91 | 34 | 1.2 | 0.87 |
| A | 31 | 14 |  |  |
| *IL27* (rs17855750) | | |  |  |
| G | 83 | 39 | 1.09 | 0.73 |
| T | 43 | 17 |  |  |

Note: *rs2609383 and rs2789975 were in complete linkage disequilibrium
